# Smoking, respiratory symptoms and likely asthma in young people: evidence from postal questionnaire surveys in the Wythenshawe Community Asthma Project (WYCAP)

**DOI:** 10.1186/1471-2466-6-10

**Published:** 2006-05-22

**Authors:** Peter Frank, Julie Morris, Michelle Hazell, Mary Linehan, Timothy Frank

**Affiliations:** 1 General Practice Research Unit, North West Lung Research Centre, Wythenshawe Hospital, Southmoor Road, Manchester, M23 9LT, UK; 2 Department of Medical Statistics, South Manchester University Hospitals NHS Trust, Wythenshawe Hospital, Southmoor Road, Manchester, M23 9LT, UK

## Abstract

**Background:**

Although it is recognised that smoking is a major risk factor for subjects with chronic obstructive pulmonary disease and is associated with respiratory symptoms, there is less agreement concerning the relationship between asthma and smoking. This study aims to examine the relationship between cigarette smoking and asthma prevalence.

**Method:**

Data were used from two postal questionnaire surveys (1999 and 2001) in two general practice populations, using a respiratory questionnaire based on the ECRHQ and a generic quality of life questionnaire (EQ-5D). Only subjects less than 45 years old were included in the survey. An empirical definition of likely asthma was used based on respiratory questionnaire responses. Smoking was examined according to three categories, current smoker, ex smoker and never smoker.

**Results:**

Almost 3500 subjects were included in the analyses. Current smokers had a higher prevalence of likely asthma compared to never smokers, odds ratio (OR) 1.59 (95% confidence interval (CI) 1.24 to 2.04). and also compared to ex smokers OR 1.79 (CI 1.25 to 2.56), but there was no difference between ex smokers and never smokers (OR 1.00 (0.75–1.35)). Current smoking was also positively associated with all symptoms but not with a history of hayfever/eczema.

**Conclusion:**

Although the positive association found between current smoking and obstructive airways disease is likely to be due to the effect of cigarettes on asthma, it could reflect an association with early COPD (GOLD stages 0 or 1). Smoking cessation has a beneficial effect on the prevalence of respiratory symptoms and is therefore of paramount importance among these young adults.

## Background

Although it is recognised that smoking is a major risk factor for subjects with chronic obstructive pulmonary disease and is associated with the symptoms of wheeze and cough [1], there is less agreement concerning the relationship between asthma and smoking [2,3].

Several studies have reported a positive association between active smoking and asthma [4-7] and some have also found a relationship between smoking cessation and asthma [8,9]. Others have found the relationship with smoking was restricted to increasing asthma morbidity [10,11]. A recent review suggested that an interaction between asthma and active cigarette smoking causes more symptoms and accelerated decline in lung function [10]. Several major studies have, however, been unable to find a significant relationship between smoking and asthma [2,12-19], although comparison of different studies is difficult due to variations in methodology and terminology, in particular the definition of asthma.

The present study aimed to examine the relationship between smoking, respiratory symptoms and likely asthma (defined by a simple and validated scoring system) in young adults using data from the Wythenshawe Community Asthma Project (WYCAP). WYCAP is a long term study examining the natural history of respiratory symptoms in two general practice populations in a deprived area of Manchester [20].

## Method

The methodology for WYCAP has been described previously in detail [20]. In summary, respiratory questionnaires were sent to all patients in the two practices in 1993, 1995, 1999 and 2001. For adults (aged 16 and over), these were based on the European Community Respiratory Health Questionnaire (ECRHQ) [21]. They included a question concerning daily cigarette consumption and two questions about exposure to environmental tobacco smoke (ETS) ("How many other adults are there in your house?" "How many of these smoke?").

In the last two surveys, an additional generic health related quality of life questionnaire, EQ-5D [22], was sent to all adult patients and this included a question concerning smoking status (current, ex, or never smoker). Ethical Approval for this study was obtained from South Manchester Local Research Ethics Committee and return of a questionnaire was taken as informed consent to participate. Only these last two surveys were used in the present analyses. In order to exclude most subjects with COPD, the analyses were restricted to patients aged between 16 and 44 years.

Subjects were categorised into three mutually exclusive groups: 1) those replying to the 1999, but not the 2001 questionnaire, 2) those replying to the 2001 but not the 1999 questionnaire and 3) those who answered both questionnaires (table 1). The data used for analysis of group 1 were from the 1999 questionnaire; for groups 2 and 3 the data used were from the 2001 questionnaire (table 1).

Likely asthma was defined as the presence of four positive responses from six key questions in the respiratory questionnaire: wheeze, woken by cough, woken by chest tightness, woken by shortness of breath (all in the previous year), a history of hay fever/eczema and family history of asthma. This simple scoring system was developed and validated for WYCAP and was found useful in identifying subjects with likely obstructive airways disease [23]. It did not differentiate asthma and COPD, but in the present study, all subjects were aged less than 45 years and so the diagnosis of established COPD was unlikely. The term 'likely asthma' has been used because a definitive diagnosis should not be made without full clinical assessment.

The associations between smoking status and the prevalence of likely asthma and of the six symptoms/risk factors included in the scoring system were examined. Smoking status used data from the EQ-5D questionnaire with subjects categorised as current, ex or never smokers. Multiple logistic regression was used to examine the effect of smoking status on the various outcome measures, after adjustment for gender, ETS (other smokers living in the house: yes/no) and age. Age was used as a continuous variable. The differences between the various categories of smoking status were expressed as odds ratios (OR) with 95% confidence intervals (CI).

## Results

Respondents in group 3, which comprised subjects who answered both the 1999 and 2001 questionnaires, were slightly older and included a greater proportion of never smokers than the other two groups (table 1). However, it was considered that no bias would be introduced by combining the three groups.

A total of 3488 subjects were included, of whom 40.7% were current smokers, 16.3% ex smokers and 43.0% never smokers. The prevalence of likely asthma was 17.2% and about one third of patients reported wheeze or being woken by cough in the previous 12 months (table 2).

After adjustment for age, gender and ETS, likely asthma was significantly less frequent among ex and never smokers compared with current smokers (ORs 0.49 (95% CI 0.37–0.65) and 0.48 (0.39–0.60) respectively) (table 2). There was no difference between ex smokers and never smokers (OR 1.00 (0.75–1.35)). The findings were similar for all the individual symptoms and also family history of asthma (table 2). Thus, the OR of wheeze for ex smokers versus current smokers was 0.40 (0.32–0.50), and it was 0.33 (0.28–0.40) for never smokers versus current smokers.

The difference between the proportions with likely asthma in current smokers and never smokers was 11.3%, which represents a simple estimate of the prevalence of 'smoking attributable' likely asthma. This estimate can be adjusted for the effects of other variables by considering the relative risk of likely asthma that is attributable to smoking as follows:

Attributable risk = frequency in never smokers × (relative risk - 1)

In the absence of a calculated relative risk, the adjusted odds ratio of 2.07 can be taken to estimate the adjusted relative risk, giving an adjusted estimate of the prevalence of 'smoking attributable' likely asthma of 13.5%.

In the case of a history of hay faver/eczema there was no significant difference between the three smoking categories (table 2).

Likely asthma was significantly less frequent in males, OR 0.74 (0.62 to 0.89) and was more common in those who reported other smokers in the house, although this finding did not reach statistical significance (OR 1.21 (1.00 to 1.46)). In this population of young adults, age had no significant effect on likely asthma prevalence (OR 0.999 (0.988 to 1.00)).

## Discussion

This study examined the relationship between smoking, respiratory symptoms and likely asthma in adults aged less than 45 years, using data from two postal questionnaire surveys carried out in 1999 and 2001. Results showed an increased prevalence in current smokers, not only compared with never smokers, but also with ex smokers. No important difference was found between ex smokers and never smokers. It is probable therefore, that smoking cessation has a beneficial effect on asthma prevalence.

The findings were similar for all the respiratory symptoms included, but no association was found between smoking status and atopy, (defined by a positive history of hay fever/eczema). Although the present results suggest that the increased prevalence of likely asthma in current smokers is related to the effect of cigarettes on individual symptoms, rather than any relationship with the atopic component of asthma, this may be an over-simplification. Our findings support recent data from the ECRHQ, which reported a negative association between smoking and sensitisation to grass pollen allergen [24]; however, the same study reported a significantly positive association between smoking and sensitisation to house dust mite allergen. An alternative, but less likely, explanation may be that the increased disease prevalence in young current smokers reflected an increase in subjects with smoking related lung disease other than asthma. This could indicate those at risk of, or with mild COPD (GOLD stages 0 or 1 respectively) [25], or those presenting with mucus hypersecretion [26]. Thus, although established COPD is less common in young adults, the disease process is likely to begin at a younger age. Recent studies have reported prevalence rates of 4–19% categorised as GOLD COPD mild, moderate or severe [27,28] and evidence of early onset of bronchial obstruction in middle aged adults has been seen both in smokers [28] and in non-smokers [27].

The findings support a recent study from Finland which reported a significant relationship between the prevalence of asthma and exposure to ETS [29], although our results did not reach statistical significance, possibly because the measure used was relatively weak, relying only on whether there were any other smokers in the house. There were not enough subjects to categorise this according to the number of other smokers; nor was there any information concerning smoking in the workplace or elsewhere.

Although cigarette smoking may modify inflammation that is associated with asthma, there are limited published data on airway pathology in smokers with asthma [10,11], and community studies concerning the relationship between smoking and asthma have given mixed results. The variations could have been due partly to differences in methodology and the varying definitions used for asthma. Several studies have included adults up to 60 years or older [8,9,12-16], thereby increasing the likelihood of confusion with COPD [3,9,12,14,15] In one study, a significant increase in self reported asthma incidence was found among patients quitting smoking compared to never smokers [13]. The conclusion was that subjects perceive COPD as asthma, and that the results might be due to misclassification rather than causality. In order to exclude cases of established COPD, the present study was limited to subjects less than 45 years old.

Currently, there is disagreement as to the exact pathophysiology of asthma and therefore it remains unclear how asthma should be defined [11]. In the present study, although the definition of likely asthma was empirical, it had the advantage of being based on the presence of four out of six key symptoms/risk factors from the questionnaire rather than relying on the single symptom of wheeze or the more subjective "physician diagnosed asthma". The effect of smoking on asthma prevalence may, however, be conservative as subjects with asthma who did not have four key features were categorised as non-asthmatics.

The 'smoking attributable' asthma prevalence was estimated in two ways, as it is possible that the use of the adjusted odds ratio as a proxy of relative risk will be an overestimate. However, the resulting estimates were very similar.

The present study was carried out in two general practice populations in a deprived area of North West England with a high prevalence of smokers [30,31], and while it is likely to represent the findings in other regions with similar socio-economic and demographic profiles; it may not be representative of the country as a whole.

The data concerning smoking status and current daily cigarette consumption were obtained from the respondents' questionnaire replies, and it is generally accepted that this would underestimate the prevalence of smoking. It seems likely that patients with a chronic lung disease such as asthma would be more likely to under-state their cigarette consumption than healthy subjects. This would strengthen our finding of a positive relationship between smoking and asthma.

## Conclusion

Although the positive association found between current smoking and obstructive airways disease is likely to be due to the effect of cigarettes on asthma, it could reflect an association with early COPD (GOLD stages 0 or 1). Smoking cessation has a beneficial effect on the prevalence of respiratory symptoms and is therefore of paramount importance among these young adults.

## Competing interests

The author(s) declare that they have no competing interests.

## Authors' contributions

PF participated in the design of the study and data analysis and drafted the manuscript. JM performed some of the statistical analyses and helped draft the manuscript. MH participated in the data analysis and helped draft the manuscript. ML helped draft the manuscript. TF participated in the design of the study and helped draft the manuscript. All authors have seen and approved the final manuscript.

## Pre-publication history

The pre-publication history for this paper can be accessed here:



## Figures and Tables

**Table 1:** Categorisation and characteristics of subjects

	**Group 1**	**Group 2**	**Group 3**
**Questionnaire(s) answered**	1999 only	2001 only	1999 and 2001
**Number of individuals**	930	1000	1558
**Age 16–24**	34.2%	41.2%	21.1%
**Age 25–34**	35.8%	31.5%	34.1%
**Age 35–44**	30.0%	27.3%	44.9%
**Female**	53.2%	51.8%	56.3%
**Never smokers**	40.0%	39.1%	47.2%
**ETS exposure**	53.8%	52.7%	46.0%

**Table 2:** Percentage prevalence of likely asthma, symptoms and risk factors (number of individuals) by smoking category and odds ratios (95% confidence interval) adjusted for gender, ETS and age

	**Current smoker**	**Ex smoker**	**Never smoker**	**Total**
**Wheeze**	44.7 (628/1405)	23.6 (134/568)	20.8 (309/1488)	30.9 (1071/3461)
**Adjusted odds ratio (95% CI)**	3.00 (2.52 to 3.56)	1.2 (0.95 to 1.52)	1	
**Cough**	43.9 (620/1411)	28.0 (158/565)	28.6 (426/1488)	34.8 (1071/3464)
**Adjusted odds ratio (95% CI)**	1.96 (1.66 to 2.30)	0.95 (0.77 to 1.19)	1	
**Shortness of breath**	14.7 (208/1411)	8.3 (47/566)	8.4 (126/1492)	11.0 (381/3469)
**Adjusted odds ratio (95% CI)**	1.74 (1.36 to 2.23)	0.94 (0.66 to 1.33)	1	
**Chest tightness**	28.2 (397/1408)	18.1 (102/565)	15.2 (227/1492)	21.0 (726/3465)
**Adjusted odds ratio (95% CI)**	2.10 (1.73 to 2.54)	1.24 (0.96 to 1.60)	1	
**History of hay fever/eczema**	32.5 (460/1414)	35.0 (199/565)	37.2 (556/1493)	35.0 (1215/3476)
**Adjusted odds ratio (95% CI)**	0.86 (0.74 to 1.02)	0.93 (0.75 to 1.14)	1	
**Family history of asthma**	61.1 (860/1408)	55.3 314/568)	54.0 (802/1486)	57.1 (1976/3462)
**Adjusted odds ratio (95% CI)**	1.30 (1.11–1.52)	1.06 (0.87–1.29)	1	
**Likely asthma**	23.9 (327/1368)	12.7 (71/560)	12.6 (184/1457)	17.2 (582/3385)
**Adjusted odds ratio (95% CI)**	2.07 (1.68 to 2.54)	1.00 (0.75 to1.35)	1	
